# Periodontitis and Cognitive Decline in Alzheimer’s Disease

**DOI:** 10.1371/journal.pone.0151081

**Published:** 2016-03-10

**Authors:** Mark Ide, Marina Harris, Annette Stevens, Rebecca Sussams, Viv Hopkins, David Culliford, James Fuller, Paul Ibbett, Rachel Raybould, Rhodri Thomas, Ursula Puenter, Jessica Teeling, V. Hugh Perry, Clive Holmes

**Affiliations:** 1 Dental Institute, Kings College London, Guy's Hospital, London, United Kingdom; 2 University of Southampton, Faculty of Medicine, Clinical Experimental Science, Southampton, United Kingdom; 3 Memory Assessment and Research Centre, Moorgreen Hospital, Southampton, United Kingdom; 4 University of Southampton, Faculty of Health Sciences, NIHR CLAHRC Wessex Methodological Hub, Southampton, United Kingdom; 5 University of Southampton, Faculty of Natural and Environmental Science, Centre for Biological Sciences, Southampton, United Kingdom; 6 Medical Research Council Centre for Neuropsychiatric Genetics and Genomics, Institute of Psychological Medicine and Clinical Neurosciences, Cardiff University, Cardiff, United Kingdom; Biomedical Research Foundation, UNITED STATES

## Abstract

Periodontitis is common in the elderly and may become more common in Alzheimer’s disease because of a reduced ability to take care of oral hygiene as the disease progresses. Elevated antibodies to periodontal bacteria are associated with an increased systemic pro-inflammatory state. Elsewhere raised serum pro-inflammatory cytokines have been associated with an increased rate of cognitive decline in Alzheimer’s disease. We hypothesized that periodontitis would be associated with increased dementia severity and a more rapid cognitive decline in Alzheimer’s disease. We aimed to determine if periodontitis in Alzheimer’s disease is associated with both increased dementia severity and cognitive decline, and an increased systemic pro inflammatory state. In a six month observational cohort study 60 community dwelling participants with mild to moderate Alzheimer’s Disease were cognitively assessed and a blood sample taken for systemic inflammatory markers. Dental health was assessed by a dental hygienist, blind to cognitive outcomes. All assessments were repeated at six months. The presence of periodontitis at baseline was not related to baseline cognitive state but was associated with a six fold increase in the rate of cognitive decline as assessed by the ADAS-cog over a six month follow up period. Periodontitis at baseline was associated with a relative increase in the pro-inflammatory state over the six month follow up period. Our data showed that periodontitis is associated with an increase in cognitive decline in Alzheimer’s Disease, independent to baseline cognitive state, which may be mediated through effects on systemic inflammation.

## Introduction

Periodontal disease is widespread in the UK population and typical of most other westernized countries including North America[1]. In older age groups it is believed to be a major cause of tooth loss. In the UK in 1998, only 38% of adults aged over 65 had 21 or more of their original 32 teeth with 50% of these reporting periodontitis before they lost teeth[2]. A number of studies show that having few teeth, possibly as a consequence of earlier periodontitis, is associated with an increase risk of developing dementia [3].

Periodontitis has been shown to be associated with a raised serum pro-inflammatory state as shown by increases in C Reactive Protein (CRP) [4] and pro-inflammatory cytokines (e.g. Tumour Necrosis Factor α (TNFα)) with a reduction in anti-inflammatory markers (e.g. interleukin 10 (IL 10)) [5]. Chewing on involved teeth may lead to the introduction of periodontal bacteria shown by detectable amounts of serum bacterial lipopolysaccharide (LPS) [6,7]. In Alzheimer’s Disease (AD) periodontitis may be even more common because of a reduced ability to take care of oral hygiene as the disease progresses and in AD increased elevation of serum levels of antibodies with associated increases in TNFα have been reported [8]. We have previously shown that chronic inflammatory diseases are associated with increased systemic pro-inflammatory cytokines and an increased rate of cognitive decline in AD [9]. We hypothesised that periodontitis would increase with increasing dementia severity in AD but that periodontitis would be associated with an increased rate of cognitive decline independent of the degree of dementia severity. We further hypothesised that periodontitis would be associated with a relative increase in systemic measures of the pro-inflammatory state and a decrease in anti-inflammatory state.

## Material and Methods

### Study design

60 non smoking community dwelling participants (and their caregivers) with mild to moderate dementia and a minimum of 10 teeth who had not received treatment for periodontitis in the past 6 months, were recruited during the period August 2012 to August 2013 from clinical referrals to memory assessment services in Southampton, United Kingdom. All participants in this study had to have capacity to give consent for themselves following United Kingdom Medical Research Council guidance http://www.mrc.ac.uk/documents/pdf/medical-research-involving-adults-who-cannot-consent/. A surrogate consent procedure was not used. As part of the assessment of capacity a trained psychiatrist explained orally and in writing the nature, duration, and purpose of the study so that the participant was aware of the potential risks, inconveniences, or adverse effects that may occur. All participants in this study were considered to understand this information; to weigh up the information and retain it for long enough to make a decision as to whether to take part. All participants communicated this decision by signing the participants information sheet and consent form. The participants capacity to consent was monitored throughout the study and any participants considered to have lost this capacity were withdrawn.

Participants fulfilled National Institute of Neurological and Communicative Disorders and Stroke-Alzheimer’s Disease and Related Disorders Association (NINCDS-ADRDA) criteria for probable or possible AD [10] with a modified Hachinski Ischaemic Scale score [11] of greater than 4 points and all had been on a stable cholinesterase inhibitor for at least 6 months. Participants were cognitively tested using the Alzheimer’s Disease Assessment Scale (ADAS-cog) [12] as the primary cognitive outcome and the standardized Mini-Mental State Examination (sMMSE) [13] as a secondary cognitive outcome. Immediately following cognitive assessment at baseline a venous blood sample was taken for CRP, the pro-inflammatory cytokine TNFα, the anti-inflammatory cytokine IL10 and for antibodies to *P*.*gingivalis*. The participants’ dental health (number of teeth and measures of periodontitis including the number of sites (6 sites per tooth) with plaque scores assessed as grade 2 (plaque identifiable without the need for a dental probe) and grade 1 (plaque identifiable using a dental probe), pocket depth in millimeters per site and the number of sites showing bleeding on probing was assessed by an accredited research dental hygienist. These assessments are all used to determine the presence or absence of periodontitis following established Centre for Disease Control/American Academy of Periodontology (CDC/AAP) case definitions [14]. The participants’ main caregiver, defined as a caregiver spending at least 10 hours per week with the participant, was interviewed to assess medical and dental history including treatment for periodontitis and medication use over the previous 6 months. The participant and main caregiver were reviewed at six months and reassessed in an identical manner.

### Standard Protocol Approvals, Registrations, and Patient Consents

This study received approval from the South and West Hampshire Local Research Ethics Committee (11/SC/0422). Written informed consent was obtained from all patients participating in the study.

### Serum inflammation, antibody and DNA assays

All blood samples were centrifuged and sera aliquotted on ice and stored at -80°C within 2 hours. Levels of CRP, TNFα and IL-10 were assayed using sandwich immunoassay multi-plex cytokine assay (Meso Scale Discovery (MSD). A protocol provided by MSD for custom assays was used with no major modifications. Serum IgG antibody titre to *P*.*gingivalis* was determined by direct binding Enzyme Linked Immunosorbent Assay. Heat killed *P*.*gingvalis* (Invivogen) was coated onto 96 well maxisorp plates at a concentration of 10^8^ cells/ml. The plates were washed five times with Phosphate Buffered Saline (PBS) 0.05% tween, and blocked with 1% PBS Bovine Serum Albumin (BSA). Patient serum samples were added at a dilution of 1:125 in PBS 1% BSA and incubated for 90 minutes. After washing five times, goat anti-human IgG (Sigma) was added at a dilution of 1:40,000 in PBS 1% BSA, and incubated for 60 minutes. Bound IgG was detected using Tetramethylbenzidine (Sigma) as a substrate, and the reaction was stopped using 1M H_2_SO_4_ before reading optical density at 450nm. Blood for DNA (principally APOE genotype) was taken at baseline. APOE genotypes were determined by Taqman genotyping of SNP rs7412 and KASP genotyping of SNP rs429358.

### Statistical analysis

Assessment of normality of continuous variables was determined by quantile-quantile plots of the residuals. Baseline ADAS-COG, sMMSE; IL10; TNFα; optical density to IgG antibodies to *P*. *gingivalis* and changes in ADAS-COG; sMMSE and TNFα were normally distributed. Baseline serum CRP; and changes in CRP and IL10 levels were not normally distributed. Baseline comparisons were made using t tests and Mann-Whitney tests for continuous variables, depending on whether normally distributed or not. For categorical variables, Chi squared tests were used. Comparisons between the two groups were made using linear regression, with adjustment for potential confounding factors of age; gender and baseline cognitive score (ADAS-COG or sMMSE). Previous studies have suggested that around half of participants in this age group would have periodontitis. Power calculations of 52 participants completing the study were based on statistical evidence to suggest that a sample size of 26 patients per group is an optimum figure for a pilot study of this nature [15,16]. Assuming a standard deviation change of 5 points this study size gave us 80% power to detect a meaningful clinical difference effect size of 4 points change in the ADAS-COG over a 6 months period (α = 0.05). The unequal ratio between groups found in the study (1.7) meant that a total of 56 subjects were needed for the same effect size and power. Allowing for a 10–20% drop out rate this required the recruitment of 57 to 65 participants. In this pilot study we made no explicit allowance for multiplicity of testing, and therefore make no claims about significance where an adjustment of the type I error rate (*e*.*g*. Bonferroni) would lead to a given p-value no longer reaching statistical significance. Multivariate analysis was used to adjust for possible confounding effects of age, gender and baseline cognitive status.

## Results

### Baseline data

Following consent procedures one participant was found to be an intermittent smoker and was excluded from further involvement in the study. Of the 59 participants remaining all fulfilled NINCDS-ADRDA criteria for probable or possible AD and all had a modified. Hachinski Ischaemic Scale score of greater than four points.

### Baseline demographics, dental health measures and cognition

The mean age of the cohort at baseline was 77.7 (s.d. 8.6) years. 30 (51%) participants were men.

89.0 (s.d. 12.5) % participants had detectable plaque of whom 19.9 (s.d. 11.8) % had plaque identifiable without the need for a dental probe (grade 1) and 69.1 (s.d. 20.6) % participants had identifiable plaque using a dental probe (grade 2). The mean probing depth was 2.5 (s.d. 0.4) mm per participant with 6.7 [IQR 2.5 to 13.6] % of sites probing greater than 3mm. 13.6 [IQR 7.6 to 21.5] % of gingival sites showed bleeding on probing. Examination of the variables for plaque; probing depth and bleeding sites resulted in the classification of 22 (37.3%) participants fulfilling CDC/AAP criteria for the presence of periodontitis of whom 15 (25.4)% had moderate and 7 (11.9%) had severe periodontitis. At baseline participants had a mean number of 21.9 (s.d. 5.2) teeth, ranging from 10 to 32 teeth present.

At baseline participants had a mean ADAS-COG score of 46.2 (s.d. 12.3) points and a mean sMMSE score of 20.4 (s.d. 5.0) points.

Table 1 shows the relationship between the presence of periodontitis and baseline demographics (age and gender); other dental measures (number of teeth and *P*. *gingivalis* antibody levels) and cognitive scores (ADAS-COG and sMMSE). Patients with periodontitis were younger and had more teeth.

**Table 1:** Relationships between the presence of periodontitis and baseline demographics, other dental health measures and cognitive scores.

Characteristics	Periodontitis present (n = 22)	Periodontitis absent (n = 37)	Mean difference (95%CI) or X^2^, p value
**Age, years (s.e)**	74.9 (2.0)	79.4 (1.3)	4.5 (0.02 to 9.0), p = 0.05
**Gender, number (% male)**	13 (59%)	17 (46%)	χ^2^ 0.9, p = 0.3
**Number of teeth, number (s.e.)**	24.6 (0.9)	20.3 (0.8)	4.3 (1.7 to 6.9), p = 0.002
***P*.*gingivalis* antibody, Units (s.e.)**	0.38 (0.02)	0.37 (0.02)	0.01 (-0.05 to 0.08), p = 0.7
**Baseline ADAS-COG, points (s.e.)**	46.8 (2.2)	45.7 (2.2)	1.1 (-5.6 to 7.7), p = 0.8
**Baseline MMSE, points (s.e.)**	19.5 (1.0)	21.0 (0.9)	1.5 (-1.2 to 4.2), p = 0.3

s.e. = standard error of mean.

### Longitudinal follow up

Fifty two of 59 (88%) participants completed follow up at 6 months. One participant died; 3 participants declined follow up and 3 participants were lost to follow up. At 6 month follow up 15 (75%) of the 20 subjects fulfilling CDC/AAP criteria for periodontitis at baseline still fulfilled these criteria whilst 30 (94%) of the 32 subjects not fulfilling these criteria at baseline continued to not fulfil these criteria. The mean change in the ADAS-cog was 2.9 (s.d. 6.6) pts. The mean change in the sMMSE score was -1.4 (s.d. 3.2) pts.

The relationship between the presence of periodontitis at baseline and cognitive decline over the six month follow up period is shown in Table 2.

**Table 2:** The relationship between the presence of periodonitis at baseline and cognitive change over the six month follow up period.

Cognitive outcome	Periodontitis present (n = 20)	Periodontitis absent (n = 32)	Mean difference (95%CI), p value
**Change in ADAS-COG, points (s.e.)**	6.1 (1.2)	0.9 (1.2)	5.2 (1.7 to 8.8), p = 0.005; *4.9 (1.2 to 8.6), p = 0.01
**Change in sMMSE, points (s.e.)**	-2.5 (0.9)	-0.7 (0.4)	-1.8 (-3.6 to -0.03), p = 0.04; *-1.8 (-3.6 to 0.04), p = 0.06

s.e. = standard error of mean.

* adjusted for baseline age, gender and cognitive score (ADAS-COG or sMMSE).

There was a significant difference in the rate of change in the ADAS-cog score and the presence or absence of periodontitis at baseline which remained following adjustment for baseline age, gender and baseline ADAS-cog score. There was also a significant relationship between the presence of periodontitis and change in sMMSE score but this was not significant when adjusting for baseline age, gender or baseline sMMSE score.

There was no significant association between the rate of decline on the sMMSE score and with the number of teeth at baseline (Pearson -0.1 p = 0.4) but there was a significant association with the rate of decline on the ADAS-cog score (Pearson 0.35 p = 0.01). However, this relationship was no longer significant when adjusting for the presence of periodontitis at baseline (adjusted correlation 0.25 p = 0.07).

There was no significant association between baseline serum *P*. *Gingivalis* antibody levels and the rate of decline on the ADAS-cog score (Pearson -0.03 p = 0.9) or rate of decline on the sMMSE score (Pearson -0.2 p = 0.1).

### Systemic inflammatory markers

54 (92%) participants gave consent for blood sampling for peripheral inflammatory markers (CRP, TNFα, IL10) and DNA for ApoE genotyping at baseline.

Baseline serum levels and relationships with baseline demographics and cognitive and dental measures are shown in Table 3.

**Table 3:** Relationships between baseline serum inflammatory markers and baseline demographics; cognitive measures and dental health measures.

Characteristics	CRP	TNFα	IL10
**Baseline measure**	1.05 [IQR 0.37, 2.47], μg/ml	2.4 (s.d. 1.1), pg/ml	0.38 (s.d. 0.23), pg/ml
**Age, yrs**			
Correlation, p value	Spearman 0.12, 0.93	Pearson 0.25, 0.08	Pearson 0.06, 0.68
**Gender**			
Male	0.65 IQR [0.35, 2.58], μg/ml	2.32 SE (0.18), pg/ml	0.37 SE (0.04), pg/m
Female	1.26 IQR [0.36,1.97], μg/ml	2.48 SE (0.24), pg/ml	0.41 SE (0.05), pg/ml
p value	0.9	0.6	0.5
**sMMSE, pts**			
Correlation, p value	Spearman -0.01, 0.95	Pearson -0.22, 0.11	Pearson 0.14, 0.31
**ADAS-cog, pts**			
Correlation, p value	Spearman 0.03, 0.8	Pearson 0.22, 0.11	Pearson -0.07, 0.61
**Periodontitis**			
Absent	1.11 IQR [0.30, 4.32], μg/ml	2.44 SE (0.16), pg/ml	0.37 SE (0.04), pg/ml
Present	0.86 IQR [0.50,1.68], μg/ml	2.33 SE (0.29), pg/ml	0.41 SE (0.05), pg/ml
p value	0.76	0.71	0.49
**Number of teeth**			
Correlation, p value	Spearman -0.03, 0.84	Pearson -0.15, 0.29	Pearson 0.03, 0.81
***P*.*gingivalis* IgG concentration**			
Correlation, p value	Spearman 0.20, 0.16	Pearson 0.23, 0.09	Pearson 0.24, 0.09

There were no significant relationships between baseline systemic inflammatory markers and demographic; cognitive or dental measures.

31 (54%) participants carried the ApoE e4 allele. There were no significant relationships between carriers of the ApoE e4 allele and dental measures including the presence or absence of periodontitis (32% participants with the e4 allele compared with 43% participants without the e4 allele had periodontitis X^2^ 0.7 p = 0.4).

43 of the 52 (83%) participants who agreed to follow up gave a blood sample for peripheral inflammatory markers (CRP, TNFα, IL10) at six month follow up. Changes in serum levels and relationships with baseline dental measures are shown in Table 4.

**Table 4:** Relationships between change in serum inflammatory markers from baseline and baseline dental health measures.

Characteristics	CRP	TNFα	IL10
**Periodontitis**			
Absent	-0.02 IQR [-0.72, 0.33], μg/ml	0.16 SE (0.11), pg/ml	0.05 IQR [-0.05, 0.12], pg/ml
Present	0.29 IQR [-0.14,1.44], μg/ml	0.03 SE (0.12), pg/ml	-0.09 IQR [-0.20,0.06], pg/ml
p value	0.08	0.42	0.047
**Number of teeth**			
Correlation, p value	Spearman 0.07, 0.65	Pearson -0.01, 0.96	Spearman -0.05, 0.77
***P*.*gingivalis* IgG concentration**			
Correlation, p value	Spearman -0.18, 0.25	Pearson 0.34, 0.03	Spearman -0.36, 0.02

No significant relationships were found with number of teeth and changes in serum inflammatory markers. However, the presence of periodontitis at baseline was associated with a fall in serum IL10 levels, and serum baseline *P*. *gingivalis* antibody levels were also associated with a fall in serum IL10 levels and an increase in serum TNFα levels.

## Discussion

A number of studies have shown that patients with AD have poorer dental health than aged controls and that the more severe the dementia the worse the dental health [17,18], most likely reflecting increasing difficulties with self care in more severe dementia [19,20]. We did not find a clear relationship between severity of dementia and degree of periodontitis, most likely reflecting the absence of subjects with severe dementia in the study. However, no studies to date have examined whether, in a longitudinal study, poor dental health correlates with poorer cognitive outcomes that are independent to baseline cognitive state. This study shows that in AD poor dental health, and in particular, the presence of periodontitis, is associated with a marked increase in cognitive decline over a six month follow up period, independent to baseline cognitive state. As anticipated baseline periodontal state appeared largely stable over the six month period, although there was some improvement in some participant’s most likely reflecting heightened awareness of the potential importance of oral care due to participation in the study.

A number of reasons might be given for the relationship between periodontitis and increased cognitive decline. Firstly, the small numbers of participants in this study cannot rule out that the relationship is due to chance and the study needs to be replicated. Secondly, it is possible that participants with a more precipitous rate of cognitive decline become more susceptible to periodontitis through an unknown mechanism that is independent to the degree of cognitive impairment or, thirdly, that periodontitis is a reflection of a confounding factor such as a compromised or modified inflammatory or immune response that is also a driver of AD progression. On the other hand, periodontitis may be a direct driver of disease progression. The presence of periodontitis is determined by the examination of sites around teeth and thus the positive relationship between periodontitis and higher number of teeth was expected. However, the lack of relationship between low tooth number, a possible indicator of past periodontitis, and cognitive decline suggests that active chronic periodontitis is most important in driving cognitive decline once AD is established. A number of studies have suggested that periodontitis is associated with higher amyloid precursor protein expression [21] and higher amyloid loads in the elderly [22]. In addition other studies have shown that antibodies to common periodontal microbiota [23,24] or tooth loss [3,25] caused by chronic periodontitis, might precede the development of AD by many years. We did not find a significant relationship between serum baseline *P*. *gingivalis* antibody levels and rates of cognitive decline. The relationship may require the additional role of other periodontal organisms or it may be that in some individuals antibody levels to *P*. *gingivalis* reflect exposure to the organism which hasn’t resulted in periodontal disease.

The mechanism for the relationship between periodontitis and cognitive decline is still unclear but there is increasing evidence to support a role for systemic inflammation. Thus, cross sectional studies have shown that the presence of periodontitis, or antibodies to common periodontal bacterial flora, are associated with an increase in a systemic proinflammatory state characterized by an increase in serum CRP; TNFα [8] and TNFα/IL10 ratios [26] in participants with AD. We have previously shown that AD patients with a range of acute and chronic inflammatory conditions have an increased pro-inflammatory cytokine profile that is associated with an increased rate of cognitive decline [9]. In this current study we show evidence of a relative increase in the proinflammatory state and decrease in the anti-inflammatory state over a six month follow up period in AD participants with periodontitis. A similar association was found between pro-inflammatory state and the presence of IgG antibodies to *P gingivalis*, generally associated with the presence of periodontitis. If, as this current study suggests, there is a direct relationship between periodontitis and cognitive decline then treatment of periodontitis might be a possible treatment option in AD. Encouragingly a small study of periodontal treatment [27], in AD participants suggest that there may be some cognitive benefits to this approach supporting the need for a randomized trial to test this hypothesis.

## Supporting Information

S1 Dataset A minimum dataset of the study. (SAV)
